# A Multiobjective Approach to Homography Estimation

**DOI:** 10.1155/2016/3629174

**Published:** 2015-12-28

**Authors:** Valentín Osuna-Enciso, Erik Cuevas, Diego Oliva, Virgilio Zúñiga, Marco Pérez-Cisneros, Daniel Zaldívar

**Affiliations:** ^1^Sciences Division, Centro Universitario de Tonalá of Universidad de Guadalajara, 45400 Guadalajara, JAL, Mexico; ^2^Electronic Division, Centro Universitario de Ciencias Exactas e Ingenierías of Universidad de Guadalajara, 44430 Guadalajara, JAL, Mexico; ^3^Computer Sciences Department, Tecnológico de Monterrey Campus Guadalajara, 45201 Guadalajara, JAL, Mexico

## Abstract

In several machine vision problems, a relevant issue is the estimation of homographies between two different perspectives that hold an extensive set of abnormal data. A method to find such estimation is the random sampling consensus (RANSAC); in this, the goal is to maximize the number of matching points given a permissible error (Pe), according to a candidate model. However, those objectives are in conflict: a low Pe value increases the accuracy of the model but degrades its generalization ability that refers to the number of matching points that tolerate noisy data, whereas a high Pe value improves the noise tolerance of the model but adversely drives the process to false detections. This work considers the estimation process as a multiobjective optimization problem that seeks to maximize the number of matching points whereas Pe is simultaneously minimized. In order to solve the multiobjective formulation, two different evolutionary algorithms have been explored: the Nondominated Sorting Genetic Algorithm II (NSGA-II) and the Nondominated Sorting Differential Evolution (NSDE). Results considering acknowledged quality measures among original and transformed images over a well-known image benchmark show superior performance of the proposal than Random Sample Consensus algorithm.

## 1. Introduction

A homography is a transformation that maps points of interest by considering movements as translation, rotation, skewing, scaling, and projection among image planes, all of them contained into a single, invertible matrix. In general terms, those displacements could be considered to be belonging to three cases: (1) an object moving in front of a static camera, (2) a static scene captured by a moving camera, and (3) multiple cameras from different viewpoints. In either case, those approximations simplify the utilization of image sequences to construct panoramic views [1–3], to increment resolution in low quality imagery [4–6], to remove camera movements when studying the motion of an object into a video [7], and to control the position of robots [8–10], among other uses [11–13].

Taking a set of experimental data as a base, in a modeling problem there exist two data types: those that can be adjusted to a model with a certain probability (also known as inliers) and those that are not related to the model (e.g., outliers). There are several algorithms specialized in solving this classification problem; one of such techniques is the Random Sample Consensus (RANSAC) [14].

In the algorithm, minimum subsets of experimental data are randomly taken, and a model is proposed and evaluated according to a permissible error (Pe), in order to determine how well the model adjusts to the data [15]. This process is repeated until a number of iterations are completed, and the model with the maximum number of inliers is taken.

Even considering that RANSAC is a robust and simple algorithm, it has some drawbacks [16–18], two of which are the high dependency between the number of matching points (model quality) and the permissible error. In this work, it is considered that those disadvantages belong to a multiobjective optimization problem. On the one hand, due to the random nature of RANSAC, achieving improvements in the quantity of inliers implies more iterations in order to discard unadjusted data to the proposed model. On the other hand, the number of matching points conflictingly depends on the permissible error (Pe). A low Pe value increases the accuracy of the model but degrades its generalization ability to tolerate noisy data (number of matching points). By contrast, a high Pe value improves the noise tolerance of the model but adversely drives the process to false detections. The main error source in the model estimation procedure arises from defining the Pe value with no consideration of the relationship between the dataset and the model.

In order to make the RANSAC algorithm more efficient, some improvements have been suggested; for instance, in the algorithm called MLESAC [19] it is considered that the inliers into the images will follow a Gaussian distribution whereas the outliers are considered as uniformly positioned; according to that, the voting process is achieved through maximizing the likelihood and the original RANSAC. The SIMFIT method [20] proposed the forecasting of the permissible error, through an iterative reestimation of that value, until the model is adjusted to the experimental data. Some other variants to the original RANSAC are the projection-pursuit method, the Two-Step Scale Estimator, and the CC-RANSAC [15, 21, 22], all of them focused on maximizing the number of inliers by making more searches into the data and therefore making the complete process more expensive, computationally speaking. In such sense, an algorithm that tries to reduce the computational cost is the one proposed in [17], where the maximization of the inliers is achieved by using a metaheuristic technique, called Harmony Search.

Nevertheless the mentioned improvements, the search strategy used in the mentioned articles (with exception of [17]), are more close to random walking, and therefore those approaches are computationally expensive. Moreover, in all the cases only one objective function is considered, usually related to the number of matching points, while the permissible error is left behind. In accordance with that, and in order to overcome the typical RANSAC problems, we propose to visualize the RANSAC operation as a multiobjective problem solved by an evolutionary algorithm. Under such point of view, at each iteration, new candidate solutions are built by using evolutionary operators taking into account the quality of the previously generated models, rather than purely random, reducing significantly the number of iterations. Likewise, new objective functions can be added to incorporate other elements that allow an accurate evaluation of the quality of a candidate model.

When an optimization problem involves more than one objective function, the procedure of finding one or more optimum solutions is known as multiobjective optimization (MO) [23]. Under MO, different solutions produce conflicting scenarios among the objectives [24]. Contrary to single objective optimization, in MO it is usually difficult to find one optimal solution. Instead, algorithms for optimizing multiobjective problems try to find a family of points known as the Pareto optimal set [25]. These points verify that there is no different feasible solution which strictly improves one component of the objective function vector without worsening at least one of the remaining ones. Evolutionary algorithms (EAs) are considered the most adequate methods for solving complex MO problems, due mainly they are many times capable of maintaining a good diversity [26], can extend to multiple populations [27], as well as can work with a variety of problems such as discrete ones [28]. Several variants of nondominated sorting as well as new methods have been proposed in recent years in order to solve problems related to feature selection [29], community detection [30], among other issues [24, 31]; however, the Nondominated Sorting Genetic Algorithm II (NSGA-II) [32] and the Nondominated Sorting Differential Evolution (NSDE) [31] are some of the most representative.

In this paper, the estimation process is considered as a multiobjective problem where the number of matching points and the permissible error (Pe) are simultaneously optimized. In order to solve the multiobjective formulation, two different evolutionary algorithms have been explored: the Nondominated Sorting Genetic Algorithm II (NSGA-II) and the Nondominated Sorting Differential Evolution (NSDE). Results considering acknowledged quality measures among original and transformed images over a well-known image benchmark show superior performance of the proposal than Random Sample Consensus algorithm on the problem being assessed, giving good results even with high outliers levels.

The remainder of the paper is organized as follows: Section 2 explains the problem of image homography considering multiple views.  Section 3 introduces the fundamentals of the RANSAC method.  Section 4 briefly explains the evolutionary approaches that are used in this paper in order to solve the multiobjective problem while Section 5 presents the proposed method.  Section 6 exhibits the experimental set and its performance. Finally, Section 7 establishes some final conclusions.

## 2. Homography between Images

For the case where two images are taken of the same scene from different perspectives, a problem consists in finding a transformation that permits the matching among the pixels belonging to both images. This denominated the image matching problem. The search of a geometric transformation is achieved by utilizing corresponding points from image pairs [33, 34], which enable forming feature vectors, also called image descriptors. Even when considering that such descriptors are not completely reliable, so they can produce erroneous results for the image matching, in this paper they are used to find the geometric relations between images by using the homography, which is explained in the next paragraphs.

Consider a set of points

(1) $$\begin{array}{c} U=\left\{\;\left(\;x_{1},x_{1}^{\prime }\;\right),\left(\;x_{2},x_{2}^{\prime }\;\right),\ldots ,\left(\;x_{M},x_{M}^{\prime }\;\right)\;\right\}, \end{array}$$

such that **x**_*i*_ = (*x*_*i*_, *y*_*i*_, 1)^*T*^ and **x**_*i*_′ = (*x*_*i*_′, *y*_*i*_′, 1)^*T*^ are the positions with respect to a given image pair.

**Figure 1:**
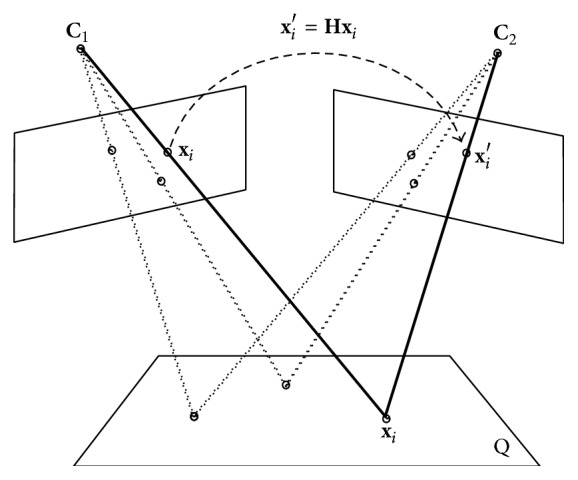
Homography from a plane between two views.

By means of a *Q* plane, a homography **H** establishes a geometric relation between two images taken under different perspectives, as can be seen in Figure 1; this allows for a projection of the points from the plane to a pair of images, through **x**_*i*_′ = **H** **x**_*i*_ or **x**_*i*_ = **H** ^−1^ **x**_*i*_′. Conducive to find the homography between an image pair, a set with four point matches is only required, to construct a linear system which must be solved [35]. Concerning evaluation of the quality of the candidate homography, it is necessary to calculate the distance among the point positions of the first image with respect to the second image; that distance is labeled as the Mismatch Error and is defined by

(2) $$\begin{array}{c} EH_{i}^{2}={\left[\;d\left(\;x_{i}^{\prime },Hx_{i}\;\right)\;\right]}^{2}+{\left[\;d\left(\;x_{i},H^{-1}x_{i}^{\prime }\;\right)\;\right]}^{2} \end{array}$$

as long as *η* = *d*(**x**_*i*_, **H** ^−1^ **x**_*i*_′) and *η*′ = *d*(**x**_*i*_′, **H** **x**_*i*_) are the respective errors from each image.

**Figure 2:**
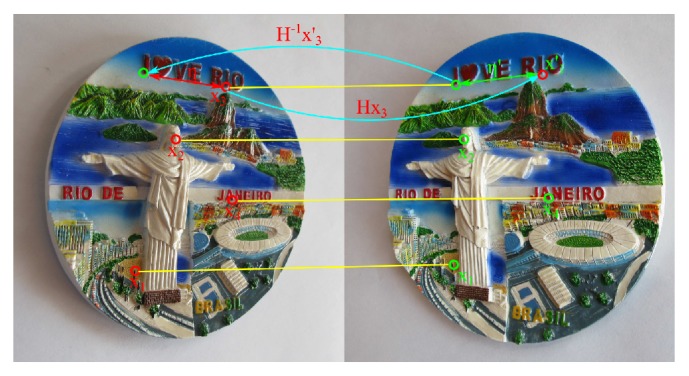
Example of evaluation process for a particular homography **H**.

Consider the example shown in Figure 2, where five correspondences **U** = {(**x**_1_, **x**_1_′),…, (**x**_5_, **x**_5_′)} are depicted; for the case of the points (**x**_3_, **x**_3_′), the error (*η*, *η*′) is considerable, and therefore the quality of the candidate homography will be ranked with a low value.

## 3. Random Sampling Consensus (RANSAC) Algorithm

To find correspondences from images through a geometric transformation (homography) and therefore to increase the number of correct matches (inliers), the use of a robust approach, such as RANSAC, is necessary. Contrary to the inliers, outliers are conflicting points related to the candidate homography.

The idea behind the algorithm consists in discovering the best hypothesis *h* ^*B*^ from a set of hypotheses *H* generated by the source data, usually corrupted with noise. The construction of candidate hypothesis *h*_*i*_ is achieved by means of a sample **S**_*i*_, with a minimum size *s*, to model estimation. As in this paper *s* = 4, then several **S**_*i*_ could be taken from the complete source data **U**, and, therefore, an exhaustive search would be computationally expensive.

In Pseudocode 1 the basic pseudocode of the RANSAC algorithms family is shown.

A subsequent step consists in finding the best candidate hypothesis *h* ^*B*^ from all the constructed and evaluated hypotheses, according to

(3) $$\begin{array}{c} h^{B}=\underset{i=1,\ldots ,G}{arg\;max\;}A_{i}\left(\;U,h_{i}\;\right). \end{array}$$

The degree of agreement is directly related to the number of inliers, and it is calculated by

(4) AiU,hi=∑j=1Mθej2hi,j=1,…,M,θej2hi=0,ej2hi>Pe,1,ej2hi≤Pe,

where Pe is a permissible error, *M* is the number of elements contained in the source data **U**, and *e*_*j*_^2^(*h*_*i*_) = *EH*_*i*_^2^ is the quadratic error produced by the *j*th data considering the hypothesis *h*_*i*_; in other words, it represents the error produced by the *i*th correspondence.

In original RANSAC algorithm, the best hypothesis is the one with the maximum number of inliers. The point *j* which produces an error *e*_*j*_^2^(*h*_*i*_) lesser than a permissible error Pe is considered as an inlier of candidate hypothesis *h*_*i*_; otherwise, it is considered as an outlier.

The RANSAC technique has to search the entire source data **U** at least once in the worst case; by considering such situation, the algorithm is similar to random walking. Several strategies could improve that kind of search, like evolutionary algorithms (EAs) [36]. These techniques are capable of exploitation and exploration of the search space judiciously, by considering that new candidate solutions will contain information regarding the best spots from search space, visited through each generation.

This work proposes working the estimation process as a multiobjective problem, by simultaneously optimizing both the number of matching points and the permissible error (Pe). In order to solve the multiobjective formulation, two different evolutionary algorithms have been explored: the Nondominated Sorting Genetic Algorithm II (NSGA-II) and the Nondominated Sorting Differential Evolution (NSDE). With the formulation, the proposed method adopts a different sampling strategy than RANSAC to generate putative solutions. Under the new mechanism, at every iteration new candidate solutions are generated based on the quality of previously found solutions, avoiding random walks in the searching process, as in the case of RANSAC.

## 4. Multiobjective Evolutionary Algorithms

A MO problem can be stated as minimizing or maximizing the function [37]

(5) fx=f1x,…,fmx,Subject  to gjx≥0,j=1,2,…,J, hkx=0,k=1,2,…,K, xiL≤xi≤xiU,i=1,2,…,n,

where a solution **x** is a vector of *n* decision variables **x** = (*x*_1_, *x*_2_,…, *x*_*n*_). The last set of constraints is called variable bounds, restricting each decision variable *x*_*i*_ to take a value within a lower *x*_*i*_^(*L*)^ and upper *x*_*i*_^(*U*)^ bound and whose limits constitute a decision space *D*. There are *J* inequalities and *K* equality constraints, both associated with the problem. In order to cover both minimization and maximization of objective functions, the operator ⊲ is used between two solutions **u** and **v**. Therefore, **u**⊲**v** denotes that solution **u** is better than solution **v** whereas **u**⊴**v** implies that solution **u** is better than or equal to solution **v**.

Different from single objective optimization, in the case of multiobjective optimization, it is usually difficult to find one optimal solution. Instead, algorithms for optimizing multiobjective problems attempt to find a group of points known as the Pareto optimal set [38]. These points verify that there is no other feasible solution which strictly improves one component of the objective function vector without worsening at least one of the remaining ones. A more formal definition of Pareto optimality or Pareto efficiency is the following.

**Definition 1 .**

If, given a solution **u**, there exists another solution **v** such that ∀*p* = 1,…, *M* *f*_*p*_(**u**)⊴*f*_*p*_(**v**) and ∃*p* ∈ {1,…, *M*} such that *f*_*p*_(**u**)⊲*f*_*p*_(**v**), then one will say that solution **u** *dominates* solution **v** (denoted by **u**≺**v**), and, obviously, solution **v** will never be sensibly selected as the solution to the problem. If *f*_*p*_(**u**)⊴*f*_*p*_(**v**), ∀*p*, one will say that solution **u** weakly dominates solution **v** and will be denoted by **u**  ⪯  **v**.

**Definition 2 .**

A solution **u** ∈ *D* is considered to be part of the Pareto optimal set *F* if and only if ∄ **v** ∈ *D* such that **v**≺**u**.

Evolutionary algorithms (EAs) are considered the most adequate methods for solving complex MO problems and some have been proposed to face such problems, where the Nondominated Sorting Genetic Algorithm II (NSGA-II) and the Nondominated Sorting Differential Evolution (NSDE) are some of the most representative.

### 4.1. Nondominated Sorting Genetic Algorithm II (NSGA-II)

NSGA-II, introduced by Deb et al. [32], is one of the most applicable and employed algorithms based on GA to solve multiobjective optimization problems. NSGA-II starts randomly generating an initial (*t* = 0) parent population *P* ^*t*^ of size *N*. During several consecutive generations (*t* = 1,…, maxIterations), the *M* objective values of *P* ^*t*^ are evaluated. Then, the population is ranked based on the nondomination sorting procedure to create Pareto optimal fronts *F*. Each individual of the population under evaluation obtains a rank equal to its nondomination level (1 is the best level, 2 is the next-best level, and so on), where the first front contains individuals with the best rank, the second front corresponds to the individuals with the second rank, and so on. In the next step, the crowding distance between members of each front is calculated by a linear distance criterion. As a binary tournament selection operator based on a crowded-comparison operator is used, it is necessary to calculate both the rank and the crowding distance of each member in the population. Using this selection operator, two members are selected among the population. Then, the member with the larger crowding distance is selected if they share an equal rank. Otherwise, the member with the lower rank is chosen. Next, a new population of offspring with a size of *N* is created using the random selection, the simulated binary crossover [19], and the polynomial mutation [20] operators to create a population consisting of the current and the new population of the size of 2*N*.

#### 4.1.1. Simulated Binary Crossover

This operator simulates the behavior of the single-point crossover on binary strings. Given as parents **x** ^(1,*t*)^ and **x** ^(2,*t*)^, they generate the *i*th component (*i* = 1,2,…, *n*) of the offspring individuals as follows:

(6) xi1,t+1=0.5·1+βi·xi1,t+1−βi·xi2,t,xi2,t+1=0.5·1−βi·xi1,t+1+βi·xi2,t,βi=2·a1/nc+1,if  a<0.5,12·1−a1/nc+1,otherwise,

where *a* is a random number in [0,1]. The parameter *n*_*c*_ determines the separation between the offspring individuals in comparison to their parents.

#### 4.1.2. Polynomial Mutation

This operator employs a polynomial distribution in the following way:

(7) xit+1=xit+xiU−xiL·δi,δi=2·a1/nm+1−1,if  a<0.5,1−2·1−a1/nm+1,otherwise,

where *x*_*i*_^(*L*)^ and *x*_*i*_^(*U*)^ are the low and upper bounds, respectively, for the *i* decision variable, whereas *n*_*m*_ represents the distribution index.

### 4.2. Nondominated Sorting Differential Evolution (NSDE)

The NSDE [31] algorithm is an extension of the original differential evolution (DE) [39] method for solving multiobjective problems. NSDE works in a similar way to DE except in the selection operation which is modified in order to be coherent with the nondominated criterion.

The algorithm begins by initializing a population of *n*-dimensional individuals and considers parameter values that are randomly distributed between the prespecified lower initial parameter bound *x*_*i*_^(*L*)^ and the upper initial parameter bound *x*_*i*_^(*U*)^. In order to generate a trial individual (solution), the DE algorithm first mutates the current individual **x**_*i*,*t*_ from the population by adding the scaled difference of two vectors from the current population:

(8) $$\begin{array}{c} v_{i,t}=x_{i,t}+F\cdot \left(\;x_{r_{1},t}-x_{r_{2},t}\;\right);\;r_{1},r_{2}\in \left\{1,2,\ldots ,N_{p}\right\}, \end{array}$$

with **v**_*i*,*t*_ being the mutant individual. Indexes *r*_1_ and *r*_2_ are randomly selected with the condition that they are different and have no relation to the individual index *i* whatsoever (i.e., *r*_1_ ≠ *r*_2_ ≠ *i*). The mutation scale factor *F* is a positive real number, typically less than one. In order to increase the diversity of the parameter element, the crossover operation is applied between the mutant individual **v**_*i*,*t*_ and the original individuals **x**_*i*,*t*_. The result is the trial individual **u**_*i*,*t*_ which is computed by considering an element to element operation as follows:

(9) $$\begin{array}{c} u_{j,i,t}=\left\{\begin{array}{cc} v_{j,i,t}, & \text{if rand}\left(0,1\right)\leq \text{CR}\;\;or\;\;j=j_{rand},\\ x_{j,i,t}, & \text{otherwise}, \end{array}\right. \end{array}$$

where *j*_rand_ ∈ {1,2,…, *D*}. The subscripts *j* and *i* are the parameter and individual indexes, respectively. The crossover parameter (0.0 ≤ CR ≤ 1.0) controls the fraction of parameters where the mutant individual is contributing to the final trial individual. In addition, the trial individual always inherits the mutant individual parameter according to the randomly chosen index *j*_rand_, assuring that the trial individual differs by at least one parameter from **x**_*i*,*t*_. Finally, a nondominated selection is used to build the Pareto optimal front. Thus, if the trial individual **x**_*i*,*t*_ dominates the target individual **x**_*i*,*t*_, the trial individual **x**_*i*,*t*_ is copied into the population for the next generation; otherwise, the target individual **x**_*i*,*t*_ is copied:

(10) $$\begin{array}{c} x_{i,t+1}=\left\{\begin{array}{cc} u_{i,t}, & \text{if }u_{i,t}≺x_{i,t},\\ x_{i,t}, & \text{otherwise}. \end{array}\right. \end{array}$$

## 5. The Proposed Approach

### 5.1. Individual Representation

In the estimation process, each candidate homography **H**_*i*_ is calculated by using four different point correspondences. The candidate homography **H**_*i*_ is thus evaluated over the entire dataset **U**, dividing all elements from the dataset to inliers and outliers, according to a permissible error (Pe).

In order to construct a candidate solution or individual **s**_*i*_, four indexes, *o*, *p*, *q*, and *r*, are selected from the set {1,2,…, *M*} of correspondences. Therefore, the homography **H**_*i*_ across the two views is computed by solving the linear system produced from the set of four point matches (**x**_*o*_, **x**_*o*_′), (**x**_*p*_, **x**_*p*_′), (**x**_*q*_, **x**_*q*_′), and (**x**_*r*_, **x**_*r*_′). Additionally, the permissible error Pe that is associated with the individual **s**_*i*_ is incorporated as a decision variable. Thus, in the proposed algorithm, an individual or candidate solution **s**_*i*_ is coded as a vector of five decision variables (**s**_*i*_ = {*s*_*i*_^1^, *s*_*i*_^2^, *s*_*i*_^3^, *s*_*i*_^4^, *s*_*i*_^5^}) that is defined by

(11) $$\begin{array}{c} s_{i}=\left[\;o,p,q,r,\text{Pe}\;\right]. \end{array}$$

In our approach, the candidate solution **s**_*i*_ presents the same functionality, that is, hypothesis *h*_*i*_ in the original RANSAC algorithm.

### 5.2. Multiobjective Problem Formulation

In the proposed approach, the estimation process is considered as a multiobjective problem where the number of matching points and the permissible error are simultaneously optimized. Under such circumstances, the multiobjective problem can be defined as follows:

(12) Maximize f1si=∑j=1Mθej2si,Minimize f2si=Pe,Subject  to 1≤o≤M, 1≤p≤M, 1≤q≤M, 1≤r≤M, 0≤Pe≤MaxE,

considering that *e*_*j*_^2^(**s**_*i*_) = [*d*(**x**_*j*_′, **H**_*i*_ **x**_*j*_)]^2^ + [*d*(**x**_*j*_, **H**_*i*_^−1^ **x**_*j*_′)]^2^ and *θ*(*e*_*j*_^2^(**s**_*i*_)) = 0, *e*_*j*_^2^(**s**_*i*_) > Pe; 1, *e*_*j*_^2^(**s**_*i*_) ≤ Pe and where Max*E* represents the maximal commensurable error produced by a candidate homography. Although Max*E* could be any high value, a sufficiently small value significantly reduces the search of Pareto fronts. In this work, Max*E* has been set to 25.

### 5.3. Computational Procedures

In order to solve the multiobjective formulation, two different evolutionary algorithms have been explored: the Nondominated Sorting Genetic Algorithm II (NSGA-II) and the Nondominated Sorting Differential Evolution (NSDE). In this section, the computational procedure of both methods is described when they face the multiobjective problem described in (12).

*(a) Nondominated Sorting Genetic Algorithm II (NSGA-II)*

(1) Generate randomly a population *P*_*t*_ of *N* five-dimensional individuals. The decision variables of each individual **s**_*i*_ (*i* ∈ {1,…, *N*}) are produced, considering a random number between their search bounds.
(2) Produce an offspring population *Q*_*t*_ from *P*_*t*_ by using simulated binary crossover and polynomial mutation.
(3) Create a new population *R*_*t*_ as the combination of *P*_*t*_ and *Q*_*t*_  (*R*_*t*_ = *P*_*t*_ ∪ *Q*_*t*_).
(4) Perform a nondominated sorting to *R*_*t*_ and identify different fronts: *F*_*j*_, *j* = 1,2,…, and so forth.
(5) Set the new population *P*_*t*+1_ = *⌀* and the counter *g* = 1.
(6) Perform *P*_*t*+1_ = *P*_*t*+1_ ∪ *F*_*g*_ and increment *g*  (*g* = *g* + 1).
(7) Assuming that |*P*_*t*+1_| represents the number of elements contained in *P*_*t*+1_, repeat step (6), until (|*P*_*t*+1_| + |*F*_*g*_| < *N*).
(8) Assuming that *w* = |*F*_*g*_|, clear the initial distance (*d*_*z*_ = 0) of each element *z* from *F*_*g*_.
(9) For each objective function *m* = 1,2,…, *M*, sort the set in worse order of *f*_*m*_. Therefore, **I** ^*m*^ = sort(*f*_*m*_, >) contains the sorted elements of the objective function *m*.
(10) For *m* = 1,2,…, *M*, assign a large distance to the boundary elements of **I** ^*m*^  (*d*_**I**_1_^*m*^_ = *d*_**I**_*w*_^*m*^_ = *∞*). For all other elements *u* = 2,3,…, *w* − 1, assign a distance calculated as follows:
  (13) $$\begin{array}{c} d_{I_{u}^{m}}=d_{I_{u}^{m}}+\frac{f_{m}\left(I_{u+1}^{m}\right)-f_{m}\left(I_{u-1}^{m}\right)}{f_{m}^{max}-f_{m}^{min}}, \end{array}$$
 where **I**_*u*_^*m*^ represent the element *u* from the sorted set **I** ^*m*^. *f*_*m*_^max^ and *f*_*m*_^min^ symbolize the maximum and minimum value of *f*_*m*_.
(11) Select the (*N* − |*P*_*t*+1_|) elements from *F*_*g*_ whose distances are the longest and include them in *P*_*t*+1_.
(12) If the maximum number of iterations has been reached, the process is thus completed; otherwise, go back to step (2).
(13) The final population *P*_*t*+1_ contains the Pareto optimal set.

*(b) Nondominated Sorting Differential Evolution (NSDE)*

(1) Set the DE parameters *F* = 0.25 and CR = 0.8.
(2) Generate randomly a population *P*_*t*_ of *N* five-dimensional individuals. The decision variables of each individual **s**_*i*_ (*i* ∈ {1,…, *N*}) are produced, considering a random number between their search bounds.
(3) Generate a trial population **T** with *N* individuals (**T** = {**t**_1_, **t**_2_,…, **t**_*N*_}) of five dimensions
 (**t**_*h*_ = {*t*_*h*_^1^, *t*_*h*_^2^, *t*_*h*_^3^, *t*_*h*_^4^, *t*_*h*_^5^}) under Procedure 1.
(4) Select the elements **s**_*i*_ (*i* ∈ {1,…, *N*}) of the next population *P*_*t*+1_ under Procedure 2.
(5) If the maximum number of iterations has been reached, the process is thus completed; otherwise, go back to step (3).
(6) The final population *P*_*t*+1_ contains the Pareto optimal set.

**Procedure 1**
**for** (*i* = 1; *i* < *N* + 1; *i*++)
**do** *r*_1_ = floor(rand(0,1) · *N*); **while** (*r*_1_ = *i*);
**do** *r*_2_ = floor(rand(0,1) · *N*); **while** ((*r*_2_ = *i*) or (*r*_2_ = *r*_2_));
*j*rand = floor(5 · rand(0,1));
    
**for** (*j* = 1; *j* < 5; *j*++) // generate a trial vector
    
**if** (rand(0,1) ≤ CR or *j* = *j*rand)
    
*t*_*i*_^*j*^ = *s*_*i*_^*j*^ + *F* · (*s*_*r*_1__^*j*^ − *s*_*r*_2__^*j*^);
    
**else**
*t*_*i*_^*j*^ = *s*_*i*_^*j*^;
**end if**
**end for**
**end for**

**Procedure 2**
**for** (*i* = 1; *i* < *N* + 1; *i*++)
**if**  (**t**_*i*_≺**s**_*i*_)
**s**_*i*_ = **t**_*i*_
     else
**s**_*i*_ = **s**_*i*_
**end if**
**end for**

## 6. Experimental Results

This part of the paper deals with several experiments performed over a collection of real images. The results exhibit the performance of NSGA-II and NSDE solving the estimation problem as a multiobjective optimization task in comparison to RANSAC. In the experiments, two performance indexes are considered: the mean squared error (MSE) and the Peak Signal to Noise Ratio (PSNR). Such indexes allow appropriately assessing the accuracy of the estimation.

The problem of homography estimation consists in finding a geometric transformation that maps points of a first view (**x**_*i*_) to a second view (**x**_*i*_′), taken from different point of view. This projective transformation **H** relates corresponding points of the plane projected into the first and second views by **x**_*i*_′ = **H** **x**_*i*_ or **x**_*i*_ = **H** ^−1^ **x**_*i*_′. In order to calculate the MSE and the PSNR, two different images are defined: the estimated image (**EI**) and the actual image (**AI**). The** EI** is produced by mapping the pixels from the first view in terms of the estimated homography **H**  (**E** **I** = **H** **x**_*i*_). On the other hand, the actual image (**AI**) corresponds to the second view image.

The mean squared error (MSE) evaluates the squared differences among the pixels of** EI** and** AI**. Considering that *D*1 × *D*2 represents the image dimensions, the MSE can be computed as follows:

(14) $$\begin{array}{c} MSE=\frac{1}{D1\cdot D2}\overset{D1}{\underset{x=1\;}{\sum }}\overset{D2}{\underset{y=1}{\sum }}{\left|\;IA\left(\;x,y\;\right)-IE\left(\;x,y\;\right)\;\right|}^{2}. \end{array}$$

The Peak Signal to Noise Ratio (PSNR) is commonly used to measure the quality of reconstruction of an image that undergoes some process. The signal in this case is the original data (**AI**), and the noise is the error introduced by the transformation** H** (**EI**). When comparing images, PSNR is an approximation to human perception of approximation quality. A higher PSNR value generally indicates that the estimation is of higher quality. PSNR is mainly defined via the mean squared error (MSE). Given *D*1 × *D*2 image, the PSNR is defined as

(15) $$\begin{array}{c} PSNR=10\cdot \log_{10}\left(\;\frac{MAX^{2}}{MSE}\;\right), \end{array}$$

where MAX is the maximum possible pixel value contained in the image.

**Figure 3:**
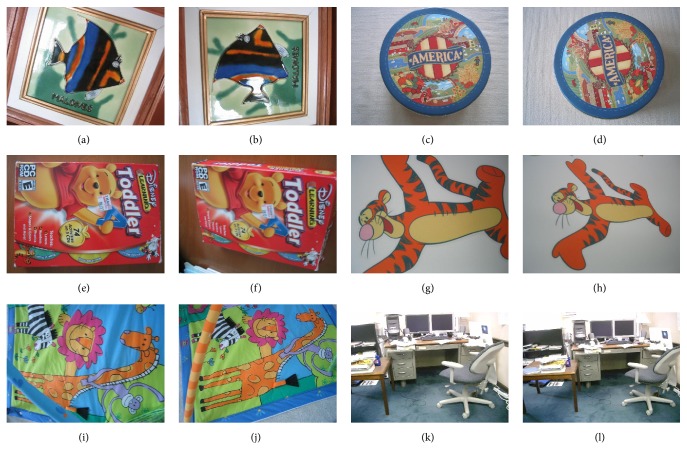
Set of images used in the experimental set.

The images used in the experiments are collected from [40] which contains several two-view images of different objects, considering a dimension of 640 × 480 pixels. Likewise, the set of images used in the experimental test is presented in Figure 3.

For the test, both algorithms, NSGA-II and NSDE, have been configured considering 200 individuals under 200 iterations. In order to conduct a fair comparison between RANSAC and multiobjective approaches, RANSAC has been operated during 40,000 iterations. Such number of calculations (200 × 200) corresponds to the maximum number of evaluations invested by NSGA-II and NSDE during their execution.

**Figure 4:**
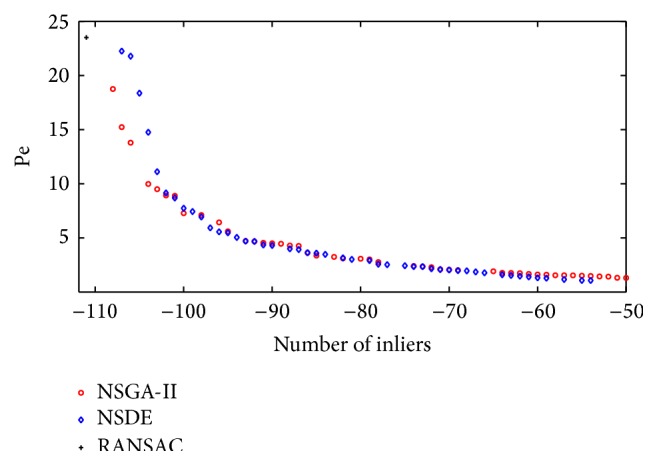
Pareto fronts found by NSGA-II and NSDE. The point obtained by RANSAC is placed only as a reference.

**Figure 5:**
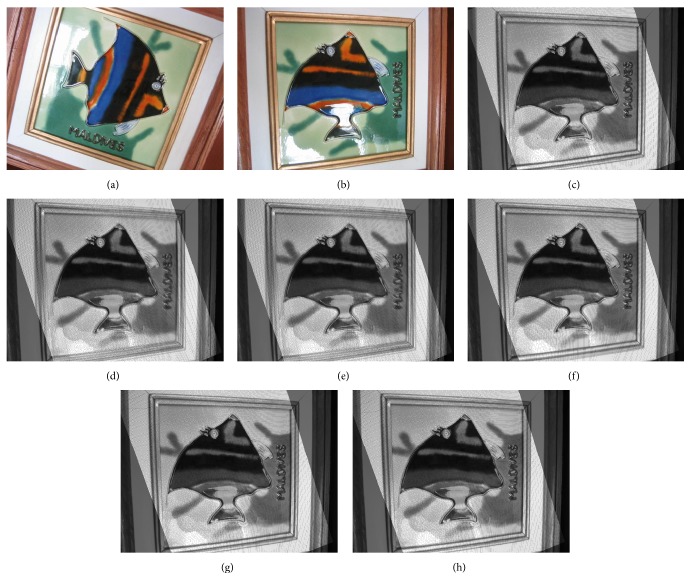
Estimation results: (a) and (b) original images, (c) and (d) RANSAC, (e) and (f) NSGA-II, and (g) and (h) NSDE.

Figure 4 shows the Pareto optimal set obtained by NSGA-II and NSDE during the estimation process, considering Figures 5(a) and 5(b) as the first and second views, respectively. In Figure 4, the best RANSAC estimations have been also included as a reference, only to validate the performance of the multiobjective approaches.

In order to illustrate the obtained results, Figure 5 shows the estimations produced by all methods in terms of their resulting estimated images. The single estimation generated by RANSAC is exhibited in Figures 5(c) and 5(d). In case of the multiobjective approaches, three solutions from the Pareto optimal set have been selected: the boundary solution for *f*_1_, the boundary solution for *f*_2_, and the median solution. Such solutions are presented in Figures 5(e)-5(f) and 5(g)-5(h), for NSGA-II and NSDE, respectively.

Table 1 presents the performance results for NSGA-II and NSDE in terms of the mean squared error (MSE) and the Peak Signal to Noise Ratio (PSNR) over three pairs of images. The results present the averaged outcomes obtained throughout 30 different executions. In order to appreciate the differences, results only report the boundary solutions obtained for *f*_1_ and *f*_2_. The best results at each experiment are highlighted in Table 1.

**Figure 6:**
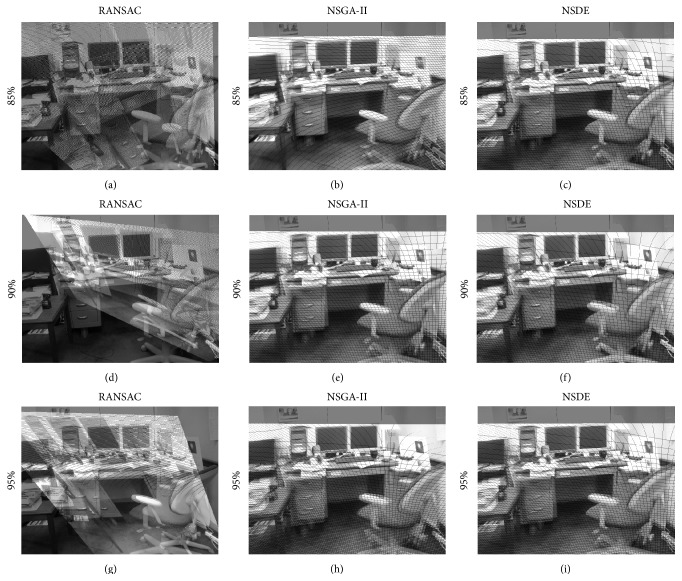
Results obtained by RANSAC, NSGA-II, and NSDE.

In order to evaluate the robustness of both algorithms, a set of outliers was added by selecting correspondence random points within the space limits. In the test, the fraction of outliers varies from 85% to 95%.  Figure 6 shows the estimations produced by all methods in terms of their resulting estimated images, considering image pairs in Figures 3(k) and 3(l).  Table 2 presents the performance results for RANSAC, NSGA-II, and NSDE in terms of the mean squared error (MSE) over the three pairs of images. The results exhibit the averaged outcomes obtained throughout 30 different executions.

From Table 2, it can be easily seen that as the number of outliers increases, the performance of each algorithm also decreases. However, the NSDE algorithm obtains the best performance in almost every case despite outliers rising above 95%.

The approach has been experimentally tested considering a set of benchmark experiments. The efficiency of the method has been evaluated in terms of the mean squared error (MSE) and the Peak Signal to Noise Ratio (PSNR) measurements. Experimental results that consider real images provide evidence on the remarkable performance of the proposed approach in comparison to the classical RANSAC.

The Wilcoxon signed rank test [41] is a nonparametric test used both to compare quantitatively some experimental data and also to determine whether there exists a meaningful difference among them. By applying the test to the data contained in Table 2, it was found that the algorithms NSGA-II and NSDE are substantially different with a 5% significance, so it can be considered that NSDE gives better results than NSGA-II when both algorithms are applied to the homography problem. In the same order of ideas, the same test was used to compare NSGA-II and RANSAC algorithms, causing the first algorithm to be better than the second.

## 7. Conclusions

In this work the use of two multiobjective evolutionary algorithms in conjunction with point correspondences is proposed to estimate homographies between image pairs. Under this approach, the estimation process is considered as a multiobjective problem with the number of matching points (*f*_1_) and the permissible error (*f*_2_) being simultaneously optimized. Under such circumstances, the approach has the capacity to find the best balance between both objectives.

A close inspection of the standard deviations from Table 1 reveals that NSGA-II maintains a big dispersion in its solutions. This aspect is mainly emphasized in the MSE index. Such an inconsistency is a consequence of the NSGA-II incapacity to produce similar solutions during its executions. On the contrary, NSDE produces better solutions than NSGA-II in terms of accuracy (MSE) and consistency. On the other hand, as a higher PSNR value indicates that the estimation is of higher quality, results produced by the NSDE algorithm exhibit the best performance.

In order to solve the multiobjective formulation, two different evolutionary algorithms have been explored: the Nondominated Sorting Genetic Algorithm II (NSGA-II) and the Nondominated Sorting Differential Evolution (NSDE).

After several tests, it was found that NSDE gives better results in solving the image matching problem presented, according to a known statistical test over a set of experimental results.

## Figures and Tables

**Pseudocode 1:**
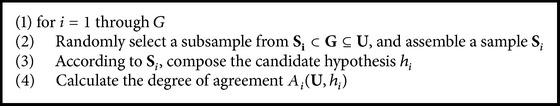


**Table 1:** Evaluation of the estimation results for NSGA-II and NSDE.

Algorithm	Image pair	MSE		PSNR	
NSGA-II	Figures 3(a) and 3(b)	97.44 (23.4474)	93.11 (19.9691)	8.59 (1.8793)	8.94 (1.6751)
NSDE		**82.64 (0.7574)**	**82.14 (0.0787)**	**9.82 (0.0791)**	**9.87 (0.0083)**

NSGA-II	Figures 3(c) and 3(d)	62.81 (20.6944)	56.09 (10.9807)	12.56 (2.4086)	13.31 (1.3887)
NSDE		**51.81 (0.1233)**	**51.87 (0.2008)**	**13.87 (0.0207)**	**13.86 (0.0336)**

NSGA-II	Figures 3(e) and 3(f)	118.23 (9.2986)	114.46 (0.4646)	6.73 (0.6693)	6.99 (0.0353)
NSDE		**114.56 (0.6316)**	**114.29 (0.2172)**	**6.98 (0.0480)**	**7.00 (0.0165)**

**Table 2:** Quality measures, calculated from single extreme values, found by RANSAC, NSDE, and NSGA-II, varying the outlier number.

Algorithm	Image pair	MSE *μ*(*σ*)		
		95% of outliers	90% of outliers	85% of outliers
NSDE	Figures 3(g) and 3(h)	**117.9057 (17.2158)**	105.3281 (3.2716)	102.7769 (1.7186)
NSGA-II		146.7161 (3.3511)	140.1202 (13.2941)	122.5373 (22.6950)
RANSAC		135.0995 (17.8106)	**103.6220 (2.8878)**	**100.8954 (1.0529)**

NSDE	Figures 3(i) and 3(j)	**70.2051 (14.8488)**	**52.0970 (0.3064)**	**51.7556 (0.1016)**
NSGA-II		91.8883 (14.9284)	85.6721 (17.6376)	61.3195 (21.0951)
RANSAC		72.2009 (25.6447)	52.1122 (0.2773)	51.8429 (0.1234)

NSDE	Figures 3(k) and 3(l)	**109.3420 (15.7526)**	95.9762 (0.4746)	**95.7580 (0.0121)**
NSGA-II		135.5036 (5.6795)	110.0551 (21.9004)	96.9498 (2.5278)
RANSAC		120.9408 (21.7259)	**95.8901 (0.1421)**	95.8631 (0.1470)
